# A single dose of neoadjuvant radiation for Merkel cell carcinoma: Complete pathologic response with minimal morbidity in a rapidly growing lesion of the eye

**DOI:** 10.1016/j.jdcr.2025.09.029

**Published:** 2025-10-10

**Authors:** Smitha Chandrasekhar, Ariel Finberg, Austin Jabbour, Luke Dang, Josiah Hanson, Sanaz Behnia, Peter Goff, Paul Nghiem

**Affiliations:** a Department of Dermatology, University of Washington Medical Center, Seattle, Washington; b Fred Hutchinson Cancer Center, Seattle, Washington; c Department of Radiology, University of Washington Medical Center, Seattle, Washington; d Department of Radiation Oncology, Seattle VA Medical Center, Seattle, Washington

**Keywords:** hypo-fractionated radiotherapy, Merkel cell carcinoma, neoadjuvant, periorbital, radiotherapy

## Introduction

Merkel cell carcinoma (MCC) is a rare skin cancer with an increasing incidence of ∼ 3000 new cases/year in the U.S. and a 40% risk of recurrence.^1^^,^^2^ Standard management for locoregional disease is excision and sentinel lymph node biopsy (SLNB), followed by radiotherapy (RT).

Single-fraction radiotherapy (SFRT) has been studied for treatment of painful/large MCC metastases with one 8 gray fraction.^3^^,^^4^ SFRT is currently being explored in both adjuvant and palliative settings. To the best of our knowledge, neoadjuvant/preoperative RT has not been described in MCC.

This case demonstrates the potential utility of neoadjuvant SFRT for MCC arising rapidly in a functionally critical location such as near the eye. This patient experienced a complete pathologic response following SFRT, highlighting the remarkable radiosensitivity of MCC, documented by surgical excision of the tumor bed 3 weeks later.

## Case report

**Fig 1:**
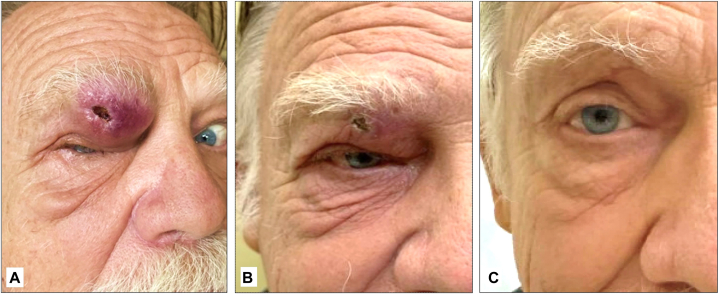
A periocular MCC before and after treatment. A Merkel cell carcinoma measuring 3.0 × 2.5 cm presented above the right eye **(A)**, obstructing vision and ability to open the eye. The central ulceration was from the prior biopsy. Eight days after SFRT **(B)**, the mass was markedly reduced in size and no longer palpable. Fourteen months after SFRT and excision of the tumor bed **(C)**. *SFRT*, Single-fraction radiotherapy.

**Fig 2:**
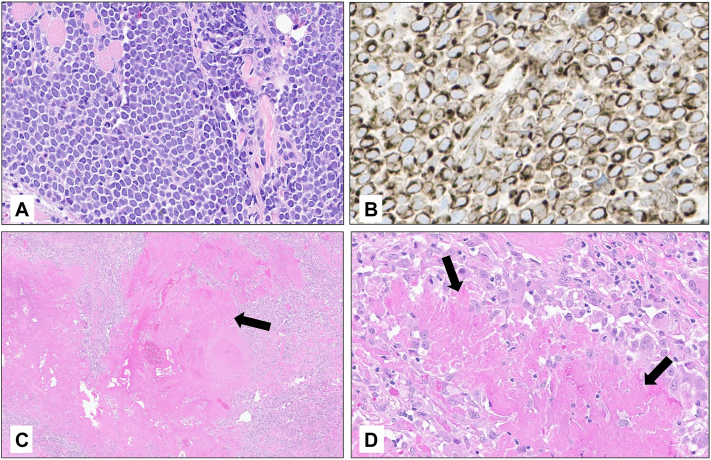
Pathology of the primary site before and after radiation. On H&E, the initial biopsy showed small round basophilic cells **(A)** which stained positive for cytokeratin 20 in a perinuclear dot-like pattern **(B)**, consistent with the diagnosis of MCC. Three weeks after SFRT, surgical excision of the tumor bed revealed necrosis (marked by *arrows*) and histiocytic inflammation **(C** and **D)**. All photos were taken with a 20× objective, except for panel **(C)** (4× objective). *MCC*, Merkel cell carcinoma; *SFRT*, single-fraction radiotherapy.

**Fig 3:**
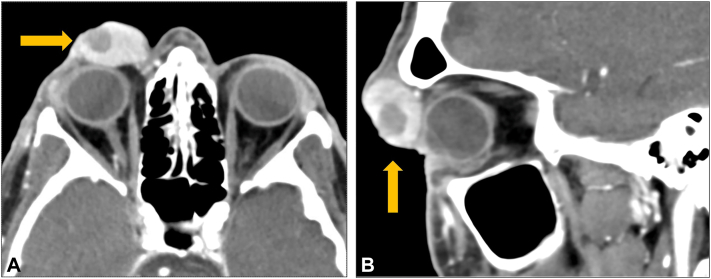
Preoperative imaging of the orbit. Coronal **(A)** and sagittal **(B)** images of a computed tomography scan of the orbit and the adjacent MCC (marked by *arrows*) that did not invade surrounding structures. A small area of central necrosis vs postbiopsy fluid was present in the tumor. *MCC*, Merkel cell carcinoma.

A 77-year-old man presented with a rapidly growing, 3 cm mass above his right eyelid (Fig 1, *A*). Immunohistochemistry was positive for CK20 in a perinuclear dot-like pattern, supporting the diagnosis of MCC (Fig 2). Antibodies to Merkel virus oncoproteins were present in the serum (titer 3760), consistent with virus-positive MCC. The patient’s baseline circulating tumor DNA (ctDNA) results were positive (18 mean tumor molecules/mL). A computed tomography scan of the orbit demonstrated tumor involvement of the subcutaneous tissues without invasion of bone or the globe (Fig 3). There was no clinical or radiographic lymphadenopathy. Urgent multi-disciplinary discussion determined that surgery could not yield clear margins without significant functional morbidity, thereby limiting its role as initial therapy. The mass had grown markedly over a few weeks and was affecting the function of his only seeing eye (left-eye blindness arose previously from an endocarditis-associated septic embolus). Thus, a decision was made to initiate SFRT promptly with the goal of temporizing the growth and ideally shrinking the tumor to a surgically addressable size.

**Fig 4:**
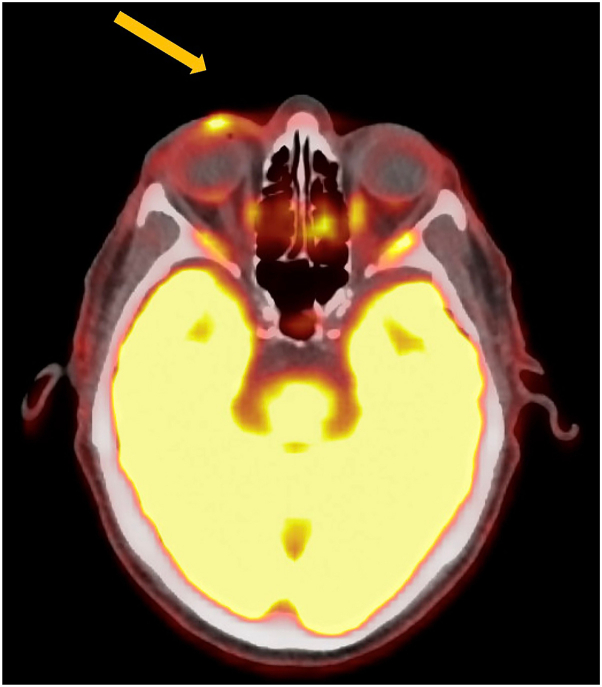
PET/CT 16 days after SFRT. The mass was no longer palpable and was markedly reduced in size, although a focus of glucose uptake (*arrow*) was present after SFRT. Eight days later, excision of this area showed no viable tumor cells. *PET/CT*, Positron emission tomography/computed tomography; *SFRT*, single-fraction radiotherapy.

Within 2 weeks, the mass was no longer palpable; however, positron emission tomography imaging showed a shrinking, but still present tumor, measuring 1.1 cm in diameter compared to the initial measurement of 3 cm, with no distant metastases on imaging (Figs 1, *B* and 4). Given the lack of published data regarding the long-term efficacy of SFRT monotherapy, as well as the initial goal of the SFRT not being curative, the decision was made to proceed with excision and SLNB. Surgical pathology of the excised tumor bed displayed necrosis and histiocytic inflammation without viable tumor cells, representing a complete pathologic response (Fig 2, *C* and *D*). After surgery, ctDNA became undetectable.

Lymphoscintigraphy identified a right submandibular node; however, intraoperatively, this could not be found. Surgery was followed by an additional dose of 8 Gy RT to the primary site and draining lymph nodes to address potential microscopic disease.

The only side effect of these treatments was grade 1 edema and erythema, which later resolved (Fig 1, *C*). At last follow up 22 months following surgery, the patient reported no subjective vision loss or changes and positron emission tomography/computed tomography imaging had remained negative for recurrent disease. Given that roughly 90% of MCC recurrences are within 2 years of treatment, the patient’s prognosis was favorable.^2^ However, ctDNA levels were intermittently positive and close follow-up was ongoing.

## Discussion

During the initial, urgent multidisciplinary conversation, many factors played into the decision to choose SFRT for initial management rather than surgery, conventional RT with definitive intent, or immunotherapy (IMTX). The importance of multidisciplinary discussion in the treatment of MCC was particularly apparent in this challenging presentation. A key consideration was that initiating treatment with either conventional RT or surgery would risk functional loss of the patient’s only seeing eye. While there have been reports of conventional RT monotherapy having minimal morbidity in MCC tumors near the orbit, the treating group’s experience felt the risks of this treatment were unnecessarily high.^5^ IMTX, a first-line option for unresectable MCC, was considered and has been studied in the neoadjuvant setting.^6^ However, IMTX has a lower likelihood of initial response (∼55%), compared to RT (95%).^6^^,^^7^ The delay to assess IMTX efficacy was felt to be particularly risky as the tumor was growing rapidly and already affecting the patient’s vision.

Emerging data show that SFRT appears to have similar rates of postoperative disease control when compared to conventional RT and has many logistical and clinical benefits.^3^^,^^4^ Iyer et al showed that SFRT had a 94% response rate in palliating large MCC tumors. Among 92 tumors, 39 had a complete clinical response; however, pathologic verification was not feasible.^7^

Although we are not aware of other reports of preoperative RT being used in MCC, this approach has been effective in many other cancer types with and without systemic therapy. Neoadjuvant RT has been shown to decrease recurrence rates and affect survival in other cancers such as rectal adenocarcinoma. The exact mechanism of its benefit is unknown, but it is evident that neoadjuvant RT can lead to better outcomes in other cancer types.^8^ To our knowledge, there are no documented cases of neoadjuvant RT for MCC, nor published data regarding the extent of pathologic response to SFRT in this setting. Although only a single case, it demonstrates that neoadjuvant RT can be well-tolerated, effective, and a potential option to reduce surgical morbidity in selected cases.^7^

SLNB is important for prognosis and management of the draining node bed in MCC. It is possible that neoadjuvant SFRT may affect the accuracy of a subsequent SLNB.^9^ Specifically, it is unclear what effect SFRT could have on small lymphatics. It is well known that higher doses of radiotherapy can damage lymphatics and cause lymphedema. In this case, a sentinel node could not be identified intraoperatively despite identification via lymphoscintigraphy. It is unclear if neoadjuvant SFRT affected lymphatic drainage in this case. However, technical SLNB failures tend to be more common in the head-and-neck area.^10^ Due to the possibility of having missed microscopic nodal involvement, the draining node bed was treated with SFRT to lower the risk of regional recurrence.^11^

While neoadjuvant RT is not described as an option in national guidelines for MCC care, it may have a role in select cases, particularly when tumors are rapidly growing and impinging on critical structures. This case was meant to highlight a nonstandard approach for MCC that resulted in significantly less morbidity than definitive surgery or conventional RT. In summary, neoadjuvant SFRT can be considered for temporizing a quickly growing tumor as it can be associated with a rapid, impressive pathologic response and reduced surgical morbidity.

## Conflicts of interest

None disclosed.
